# Redox Genetic Risk Score and the Incidence of End-Stage Kidney Disease in People with Type 1 Diabetes

**DOI:** 10.3390/cells11244131

**Published:** 2022-12-19

**Authors:** Kamel Mohammedi, Michel Marre, Samy Hadjadj, Louis Potier, Gilberto Velho

**Affiliations:** 1 Centre Hospitalier de Bordeaux, Department of Endocrinology, Diabetes and Nutrition, University Hospital of Bordeaux, 33604 Pessac, France; 2 Population Health Research Institute, McMaster University and Hamilton Health Sciences, Hamilton, ON L8S 4L8, Canada; 3 Institut Necker-Enfants Malades, INSERM, Université de Paris, 75013 Paris, France; 4 Clinique Ambroise Paré, 92200 Neuilly-sur-Seine, France; 5 Institut du Thorax, INSERM, CNRS, UNIV Nantes, CHU Nantes, 44109 Nantes, France; 6 Service d’Endocrinologie Diabétologie Nutrition, Hôpital Bichat, AP-HP, 75013 Paris, France

**Keywords:** type 1 diabetes, diabetic kidney disease, end-stage kidney disease, polymorphism, genetic risk score, oxidative stress

## Abstract

End-stage kidney disease (ESKD) is a multifactorial condition influenced by genetic background, but the extent to which a genetic risk score (GRS) improves ESKD prediction is unknown. We built a redox GRS on the base of previous association studies (six polymorphisms from six redox genes) and tested its relationship with ESKD in three cohorts of people with type 1 diabetes. Among 1012 participants, ESKD (hemodialysis requirement, kidney transplantation, eGFR < 15 mL/min/1.73 m^2^) occurred in 105 (10.4%) during a 14-year follow-up. High redox GRS was associated with increased ESKD risk (adjusted HR for the upper versus the lowest GRS tertile: 2.60 (95% CI, 1.51–4.48), *p* = 0.001). Each additional risk-allele was associated with a 20% increased risk of ESKD (95% CI, 8–33, *p* < 0.0001). High GRS yielded a relevant population attributable fraction (30%), but only a marginal enhancement in c-statistics index (0.928 [0.903–0.954]) over clinical factors 0.921 (0.892–0.950), *p* = 0.04). This is the first report of an independent association between redox GRS and increased risk of ESKD in type 1 diabetes. Our results do not support the use of this GRS in clinical practice but provide new insights into the involvement of oxidative stress genetic factors in ESKD risk in type 1 diabetes.

## 1. Introduction

Diabetic kidney disease (DKD) is one of the most common and severe complications of type 1 diabetes, and the leading cause of end-stage kidney disease (ESKD) worldwide. ESKD is a multifactorial condition involving a series of modifiable and non-modifiable determinants, including glycemic and hemodynamic factors, as well as a genetic background. Despite more intensive treatments of diabetes and hypertension, and an initial decrease in ESKD in recent decades, the risk of ESKD continues to be challenging, especially in people with long-standing type 1 diabetes [1,2]. Recent studies have shown that the incidence rate of ESKD was almost six-times higher among people with diabetes than in those without diabetes [3,4]. ESKD is a life-threatening complication in patients with type 1 diabetes, resulting in reduced quality of life, high rates of cardiovascular disease and mortality, and increased medical costs [5,6,7,8].

Epidemiological investigations and clinical observations on the familial clustering and heritability in DKD have highlighted an underlying genetic susceptibility. Despite the extensive research, the genetic architecture of DKD is poorly understood [9,10,11]. Candidate gene association analyses and genome-wide association studies (GWAS) have successfully identified numerous loci implicating many genes in the risk of DKD, but fewer investigations have examined the genetic risk of ESKD in individuals with type 1 diabetes [12,13,14,15,16]. We have previously reported associations between prevalent and incident diabetic nephropathy and allelic variations in genes associated with redox status biology, encoding for catalase (*CAT*), glutathione peroxidases (*GPX1* and *GPX4*), cytosolic (*SOD1*), mitochondrial (*SOD2*) and extracellular superoxide dismutase (*SOD3*, unpublished data), and the regulatory subunit p22phox of NADPH oxidase (*CYBA*) [17,18,19,20,21,22]. However, the combined effect of these allelic variations on the long-term risk of major kidney outcomes has not yet been studied in people with type 1 diabetes. We have hypothesized that incorporating those genetic findings as a single redox score may serve to identify individuals at high risk for major adverse kidney outcomes. Hence, we built a redox genetic risk score (GRS) based on our previous genetic associations data, and then tested the relationship between this redox GRS and the risk of ESKD in people with long-standing type 1 diabetes.

## 2. Materials and Methods

### 2.1. Study Population

We investigated data from three French and Belgian prospective cohorts designed to investigate biochemical and genetic determinants of DKD in people with type 1 diabetes [23,24,25]. This investigation was conducted according to the principles expressed in the Declaration of Helsinki. Study protocols were approved by the ethics committee of Angers University Hospital (Angers, France), and all participants from the three cohorts gave written informed consent. As previously published, SURGENE was a single center study of all volunteer individuals with type 1 diabetes attending the clinic of diabetology at Angers university hospital in France [24]. From 1989 to 1996, participants were selected on the basis of a diagnosis of type 1 diabetes before the age of 40 years, and duration of diabetes more than three years. Patients with ESKD or other chronic disease were not included in this study. The “Génétique de la Néphropathie Diabétique” (GENEDIAB) study was conducted in 17 diabetic clinics in France and Belgium [23]. Participants were recruited from May 1994 to April 1995 based on their history of type 1 diabetes diagnosed before the age of 35 years, duration of diabetes more than five years, with a past or present history of pre-proliferative or proliferative diabetic retinopathy requiring laser panphotocoagulation. Patients with cancer at terminal stage or those with personal disability were not included in this cohort. GENESIS France-Belgique study (Genetics Nephropathy and Sib Pair Study) was a family-based cohort including probands with type 1 diabetes diagnosed for five years or more [25]. Participants were recruited from November 1998 to December 2000 on the basis of the history of type 1 diabetes (diagnosis before the age of 35 years, with initial ketosis, and requirement for permanent insulin treatment within one year of the diagnosis) and past or present diagnosis of diabetic retinopathy. Participants were followed until death, or the latest study visit up to 31 May 2019. Characteristics of participants in each cohort have been previously published [26]. To maximize our study power, data from the three cohorts were pooled together for the current analysis. Secondary analyses in each single cohort were also performed (see below).

### 2.2. Study Flow-Chart

Among 1347 patients included in the three cohorts, we excluded participants with a baseline history of ESKD (hemodialysis, peritoneal dialysis, or kidney transplantation, *n* = 82), those for whom no genotyping was available (*n* = 51) and those without follow-up data regarding ESKD (59 deaths and 143 loss of follow-up, Supplemental Figure S1). Hence, 1012 participants were included in the present study. We checked that minor allele frequencies of the investigated SNPs were not different in excluded participants versus those who remained in the analysis (Supplemental Table S1).

### 2.3. Standard Biological Assessments, Clinical Conditions and Study Outcome 

Urinary albumin concentration (UAC) and serum creatinine were measured centrally at baseline using nephelometry and derivation of Jaffé’s (with adjustment to the enzymatic method when it was introduced in routine practice) methods, respectively. Estimated glomerular filtration rate (eGFR) was computed using the Chronic Kidney Disease Epidemiology Collaboration equation. UAC was categorized at baseline as normoalbuminuria (<30 mg/L), microalbuminuria (30 to <300 mg/L) and macroalbuminuria (≥300 mg/L). eGFR data was categorized at baseline as < and ≥60 mL/min/1.73 m^2^. Diabetic retinopathy was staged at baseline as absent, non-proliferative and proliferative. The definitions of other conditions were previously reported [26]. 

The study outcome was the occurrence during follow-up of new cases of ESKD, defined as the requirement of hemodialysis or kidney transplantation, or eGFR < 15 mL/min/1.73 m^2^, among participants without a history of ESKD at baseline.

### 2.4. DNA Genotyping and Genes Selected for the Redox Genetic Risk Score

Based on our previous investigations [18,19,20,21,22], we selected six SNPs from six genes (encoding for proteins involved in redox status biology) to be included in the redox GRS [18,19,20,21,22]: *CAT* rs2420388, *GPX1* rs9818758, *GPX4* rs713041, *CYBA* rs11076692, *SOD2* rs4880 and *SOD3* rs2270224 (unpublished data). *SOD1* rs17880135 was not included in the score as only few incident ESKD (*n* = 12) occurred during follow-up in participants carrying the minor allele. We assumed the impact of independent variants to be additive on the risk of ESKD. We computed a redox GRS for each individual by summing all risk alleles from the six selected SNPs, and thus the score could range from 0 to 12 risk alleles. Genotypes were determined by competitive allele-specific PCR genotyping system assays (KASP, LGC Genomics, Hoddeston, UK). Genotyping success rate was >95%. Genotyping was repeated in 5–10% of subjects with 100% concordance. All genotypes were in Hardy–Weinberg equilibrium.

### 2.5. Statistical Analyses

Categorical variables were summarized as counts with percentages and compared using chi-square statistics. Continuous variables were expressed as mean ± SD, or median (25th and 75th percentiles) for those with skewed distribution and compared using ANOVA or Wilcoxon/Kruskal–Wallis tests. Participants were categorized into three groups of equal size corresponding to increasing tertiles (T1, T2 and T3) of redox GRS.

Kaplan–Meier curves were plotted to evaluate survival rates without incidence of ESKD during follow-up by tertiles of redox GRS. Survival curves were compared using the log-rank test. Cox proportional hazards regression models were fitted to estimate hazard ratio (HR), with associated 95% confidence interval (CI), for the risk of ESKD during follow-up according to redox GRS (T2 versus T1 and T3 versus T1). The Cox analyses were adjusted for cohort membership, sex and age at baseline (model 1), plus a series of relevant confounding variables at baseline: duration of diabetes, systolic blood pressure, HbA1c, eGFR, UAC, diabetic retinopathy stages (absent, non-proliferative, proliferative), use of angiotensin-converting enzyme (ACE) inhibitors or angiotensin receptor blockers (ARBs) drugs and history of tobacco smoking (model 2). The Schoenfeld residuals method was used to check the proportional hazards assumption for the investigated association (*p* = 0.13).

We also evaluated the association of redox GRS, as a continuous variable, with the risk of ESKD using restricted cubic splines with knots at 2, 6, 8, and 10, and a reference value at 4. The HR for ESKD associated with each single additional risk allele was also computed in the whole study and in each individual cohort. We tested the interaction between cohort membership and redox GRS in their association with ESKD, by including multiplicative interaction term in the regression model. We also computed sub-distribution hazard ratios (SHR), and related 95% CI, for risk of ESKD during follow-up while accounting for the competing risk of all-cause death (further to adjusting for model 2) using Fine and Gray method [27]. 

Harrell’s c-statistics, assessed in the survival analyses, were used to evaluate the performance of the redox GRS in stratifying ESKD beyond clinical risk factors (model 2) [28]. Model calibration was assessed using the Groennesby and Borgan test. Finally, we calculated the population attributable fraction (PAF), with associated CI, of the redox GRS using the method of Newson in a survival model [29]. To reduce the likelihood of type I error, correction for multiple comparisons was performed using Bonferroni method. Thus, *p* < 0.008 was considered as significant. Statistics were performed using Stata 15 software (StataCorp, College Station, TX, USA).

## 3. Results

### 3.1. Characteristics of Participants at Baseline 

Among 1012 participants, 453 (44.8%) subjects were women, and 32%, 27% and 41% from SURGENE, GENEDIAB and GENESIS, cohorts respectively. Age, duration of diabetes, HbA1c, systolic and diastolic blood pressure were, at baseline (mean ± SD), 39.8 ± 12.8 years, 23.0 ± 11.4 years, 8.8 ± 1.8%, 131 ± 18 and 76 ± 11 mmHg, respectively (Table 1). The median UAC was 13 (25th and 75th percentiles 5, 80) mg/L and eGFR was (main ± SD) 90 ± 28 mL/min/1.73m^2^. Normoalbuminuria, microalbuminuria, macroalbuminuria and eGFR < 60 mL/min/1.73 m^2^ were present at baseline in 64%, 20%, 16% and 14% of participants, respectively.

### 3.2. Redox Genetic Risk Score

Overall, the main ± SD redox GRS was 6.4 ± 2.1 and the median (25th, 75th percentiles) was 6 (5, 8) and 4.1 ± 1.4, 6.5 ± 0.5, and 8.7 ± 1.0 in the first, second and third tertiles, respectively. Characteristics of participants were comparable across tertiles of the redox GRS, except for macroalbuminuria and eGFR < 60 mL/min/1.73 m^2^, which were more frequent in the upper versus the lowest tertiles. 

### 3.3. Incidence of ESKD

New cases of ESKD occurred in 105 (10.4%) participants during a median duration of follow-up of 14 (25th and 75th percentiles 6, 19) years, corresponding to 13,517 person-years and an incidence rate of 7.8 (95% CI, 6.4–9.4) per 1000 person-years. Characteristics of participants with or without incident ESKD are shown in Supplemental Table S2. Briefly, participants who developed ESKD during follow-up, compared with those who did not, had higher systolic and diastolic blood pressure, HbA1c and AUC, and a lower eGFR. They were also more likely to have a history of tobacco smoking, proliferative retinopathy, and to use ACE inhibitors or ARBs or any antihypertensive drugs (Supplemental Table S2).

### 3.4. Redox Genetic Risk Score and Risk of ESKD

**Figure 1:**
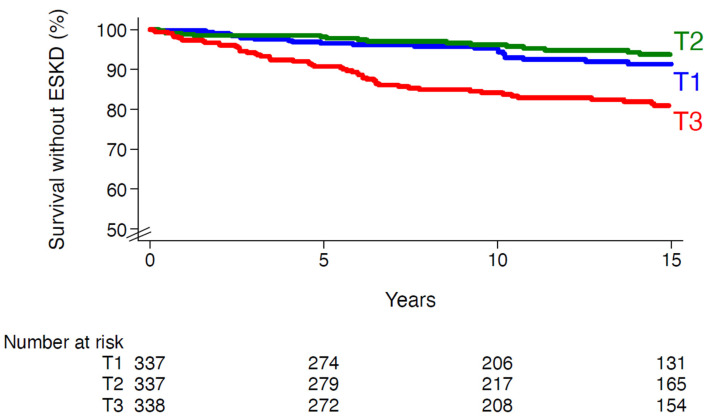
Survival without end-stage kidney disease according to tertiles of redox genetic risk score. T1, first tertile; T2, second tertile; T3, third tertile. Log-rank *p*-value < 0.0001.

Redox GRS was higher in people with versus without incidence of ESKD: 7.2 ± 2.2 versus 6.3 ± 2.1 (*p* = 0.0001). The cumulative incidences (6.8%, 6.2% and 18.1% in T1, T2 and T3, respectively, Figure 1) and incidence rates were higher in the upper tertiles than in the lowest ones (5.4, 4.5 and 13.5 per 1000 person-years in T1, T2 and T3, respectively). The relative risk of ESKD was significantly higher in the upper versus the lowest tertiles of redox GRS: HR (95% CI) for T3 versus T1: 2.87 (1.77–4.65), *p* < 0.0001 (after adjusting for cohort membership, sex and age). The magnitude of the association did not change after adjusting for additional confounding variables, including key established risk factors for DKD (model 2, Table 2). When compared with participants in T1 and T2 (considered together as a single group), those in the third tertile had an HR of 2.21 (1.44–3.42, *p* < 0.0001 adjusted for model 2). Similar results were observed when redox GRS was fitted as a continuous variable (Supplemental Figure S2). The association between redox GRS and ESKD appeared to be log-linear in individuals with a score above 4. Each additional risk allele was associated with 20% (95% CI, 8–33) increased risk of ESKD (*p* < 0.0001), after adjustment for model 2 (Table 3). Similar patterns were seen when we considered each single cohort individually (Table 3), without evidence for interaction between redox GRS and cohort membership in the associations with ESKD (*p* for interaction = 0.49). The GRS–ESKD association remained significant when we considered all-cause death as a competing risk (SHR 1.19, [95% CI 1.07–1.32], *p* = 0.001), further to adjusting for model 2. 

The PAF of the upper tertile of redox GRS on incident ESKD was estimated at 30% (95% CI, 17–42) after adjustment for relevant confounders (model 2). Notably, the estimated PAF for albuminuria (UAC > 30 mg/L) and eGFR < 60 mL/min/1.73 m^2^ were 77% (60–86) and 59% (50–67), respectively.

### 3.5. Discrimination of ESKD Risk

When conventional clinical risk factors for DKD (as in model 2) were entered in the model, the Harrell C-statistic index for risk of ESKD approximated 0.921 (95% CI, 0.892–0.950), and the redox GRS increased the discrimination only marginally to 0.928 (0.903–0.954), which represents a change in the area under curve of 0.007 (0.001–0.014, *p* = 0.04). We observed a good calibration during internal validation by using the model with both clinical risk factors and GRS (*p* = 0.12). Of note, baseline eGFR and UAC alone offer a C-statistic index of 0.896 (0.858–0.934), while the model including redox GRS together with clinical risk factors without UAC and eGFR yielded a C-statistic index of 874 (0.846–0.902). 

## 4. Discussion

In the current study, we developed a redox GRS and investigated its association with a 14-year risk of ESKD in three prospective cohorts of people with long-standing type 1 diabetes. We observed a higher risk of ESKD in participants in the top tertile of redox GRS compared with those in the lowest one. The relative risk of ESKD increased in a log-linear fashion for each additional risk allele above 4. Hence, each additional risk allele was associated with a 20% (95% CI, 8–33) increased risk of ESKD. The association was independent of relevant confounders, including baseline diabetes duration, HbA1c, systolic blood pressure, eGFR, UAC and use of ACE inhibitors or ARBs drugs. We did not observe evidence for competing risk of all-cause death in the association between redox GRS and incident ESKD. The population attributable fraction analysis suggests that 30% (95% CI, 17–42) of the ESKD hazard observed in our study was attributable to being in the upper tertile of redox GRS. However, the addition of redox GRS over usual clinical factors for DKD did not substantially increase the prediction of ESKD, which should limit its use in clinical practice. Of note, UAC and eGFR alone have excellent performances in discriminating ESKD in our cohorts (C-statistic index 0.921, 95% CI, 0.892–0.950), and it seems hard for genetic score to outperform the current screening methods in advanced DKD. However, the advantage of GRS is that it can be assessed at any time, far before the occurrence of clinical risk factors and the development of DKD. GRS could be useful, earlier in the course of the disease, in assessing the risks/benefits of preventive and therapeutic strategies.

As far as we know, our study is the first report of an association between a redox GRS and risk of ESKD in people with long-standing type 1 diabetes. Prior GWAS have identified multiple loci associated with DKD, but only a few studies have suggested a polygenic risk score (PRS) to predict ESKD, mainly in people with type 2 diabetes or the general population [30,31,32]. Yu and coworkers have recently reported a PRS in multiethnic meta-analysis of CKDGen Consortium GWAS and UK Biobank GWAS [32]. The authors observed an association between an eGFR PRS and a spectrum of incidences of kidney disease including ESKD in the Atherosclerosis Risk in Communities (ARIC) study (including 8.6% prevalent diabetes at baseline). In a type 2 diabetes setting, a GWAS PRS yielded a modest enhancement of ROC areas when added on top of clinical score (change from 0.75 (0.72–0.78) to 0.78 (0.75–0.81)) to predict diabetic nephropathy (defined as eGFR < 60 mL/min/1.73 m^2^ or a positive proteinuria dipstick) in the Chinese population [30]. The SNPs used in our genetic score did not emerge as relevant variants associated with DKD traits in GWAS among populations of type 1 diabetes [12,13,14,15,16,32]. In addition to the difference in approach used to select SNPs in our work and others (candidate genes vs. GWAS), DKD has a more complex genetic architecture than was anticipated, which could explain the lack of consistency between different GRS and limit their use to predict the individual risk of ESKD over standard risk factors in clinical setting. Additionally, uncertainties remain in our current understanding of the penetrance of genetically linked DKD. Since genetic susceptibilities interact in concert with the environment, there remains a great need to further understand the effect of environmental factors on the risk of ESKD, and to develop methods to incorporate these additional risk factors into genetic risk models. The other issue is related to the characterization of the DKD phenotypes, which is one of the main challenges for genetic research on diabetes and its complications. Unlike the studies cited above, we investigated here the 14-year risk of ESKD among people with long-standing type 1 diabetes at high risk of DKD. Future studies should investigate cohorts of people with long-standing type 1 diabetes including high quality phenotypic data and homogenous outcomes.

The key strength of the present investigation is the collection of a comprehensive set of demographic, clinical, and biological features in three multicenter, binational cohorts of middle-aged individuals with long-standing type 1 diabetes followed prospectively for a median of 14 years, with pre-specified renal outcomes. Our work has limitations to acknowledge. First, we built a GRS with a low number of SNPs from six genes selected by candidates approach rather than GWAS. In fact, our study serves as a proof of principle that a hypothesis-driven selection based on candidate gene association studies (deductive approach) may be useful to identify type 1 diabetes people at high risk for ESKD. Second, we lack external validation of our findings in other populations. Nevertheless, we investigated here three independent cohorts with consistent observations in each single cohort considered individually, without any evidence for significant interaction between cohort membership and redox GRS in their association with incidences of ESKD, which supports the intercohort replication of our findings. Third, our data may not be generalizable to all populations of type 1 diabetes since participants in our cohorts had a long duration of diabetes at inclusion with a history of diabetic retinopathy (any stages in GENESIS, pre-proliferative or proliferative in GENEDIAB), and 41% had a history of diabetic nephropathy. Finally, our findings may not be applicable to other populations from different ancestral backgrounds as our cohorts enrolled predominantly European descent.

In conclusion, this is the first report of an independent association between redox GRS and excess risk of ESKD over 14 years of follow-up in three cohorts of people with long-standing type 1 diabetes. The score yielded a relevant population attributable risk, but it does not substantially increase the ESKD prediction over standard clinical risk factors, which may limit its broad use in clinical setting. Our findings provide further insight into the involvement of genetic factors and oxidative stress into the pathophysiological mechanisms responsible for ESKD, and open new perspectives to build a widely used score based on a hypothesis-driven approach. Further genetic studies are needed to continue to enrich our understanding of DKD pathogenesis in people with type 1 diabetes, improving its prediction and flagging the way for potential molecularly targeted preventive or therapeutic interventions.

## Figures and Tables

**Table 1:** Characteristics of participants at baseline according to tertiles of redox genetic risk score.

		Tertiles of Redox GRS			*p*
	All	First	Second	Third	
N (%)	1012 (100)	337 (33.3)	337 (33.3)	338 (33.4)	
Cohort membership, *n* (%)					0.009
SURGENE	327 (32.3)	90 (26.7)	121 (35.9)	116 (34.3)	
GENEDIAB	273 (27.0)	84 (24.9)	90 (26.7)	99 (29.3)	
GENESIS	412 (40.7)	163 (48.4)	126 (37.4)	123 (36.4)	
Women, *n* (%)	453 (44.8)	155 (46.0)	157 (46.6)	141 (41.7)	0.38
Age, years	39.8 ± 12.8	40.0 ± 12.9	40.6 ± 12.2	38.8 ± 12.4	0.16
Duration of diabetes, years	23.0 ± 11.4	23.2 ± 11.2	23.5 ± 11.7	22.2 ± 11.3	0.33
Body mass index, kg/m^2^	24 ± 3	24 ± 4	24 ± 4	24 ± 3	0.39
Systolic blood pressure, mmHg	131 ± 18	130 ± 18	131 ± 17	132 ± 20	0.51
Diastolic blood pressure, mmHg	76 ± 11	75 ± 11	75 ± 10	77 ± 11	0.07
HbA1c, %	8.8 ± 1.8	8.7 ± 1.7	8.9 ± 1.7	8.9 ± 2.1	0.22
HbA1c, mmol/mol	73 ± 20	71 ± 19	73 ± 18	74 ± 23	
eGFR, ml/min/1.73 m^2^	90 ± 28	91 ± 26	91 ± 25	88 ± 32	0.19
Categories of eGFR, *n* (%)					0.001
≥60 mL/min/1.73 m^2^	871 (86.1)	300 (89.0)	299 (88.7)	272 (80.5)	
<60 mL/min/1.73 m^2^	141 (13.9)	37 (11.0)	38 (11.3)	66 (19.5)	
UAC, mg/L	13 (5, 80)	12 (5, 63)	11 (5, 41)	15 (6, 275)	0.001
Categories of UAC, *n* (%)					<0.0001
Normoalbuminuria (<30 mg/L)	651 (64.3)	221 (65.6)	237 (70.3)	193 (57.1)	
Microalbuminuria (30 to <300 mg/L)	197 (19.5)	66 (19.6)	69 (20.5)	62 (18.3)	
Macroalbuminuria (≥300 mg/L)	164 (16.2)	50 (14.8)	31 (9.2)	83 (24.6)	
Use of ACE inhibitors or ARBs, *n* (%)	322 (31.9)	110 (32.6)	97 (28.8)	115 (34.3)	0.29
Use of any antihypertensive drug, *n* (%)	399 (39.5)	139 (41.3)	116 (34.4)	144 (43.0)	0.06
Use of lipid-lowering drugs, *n* (%)	37 (3.7)	13 (3.9)	18 (5.3)	6 (1.8)	0.10
Tobacco smoking, *n* (%)					0.18
Former smokers	101 (10.0)	33 (9.8)	29 (8.6)	39 (11.5)	
Current smokers	199 (19.7)	76 (22.6)	54 (16.0)	69 (20.4)	
History of diabetic retinopathy, *n* (%)					0.16
Absent	202 (20.0)	58 (17.2)	77 (22.9)	67 (19.8)	
Non-proliferative	415 (41.0)	142 (42.1)	144 (42.7)	129 (38.2)	
Proliferative	395 (39.0)	137 (40.7)	116 (34.4)	142 (42.0)	
History of myocardial infarction, *n* (%)	36 (3.6)	14 (4.2)	11 (3.3)	11 (3.3)	0.77
History of stroke, *n* (%)	18 (1.8)	5 (1.5)	4 (1.2)	9 (2.7)	0.31
Redox genetic risk score	6.4 ± 2.1	4.1 ± 1.4	6.5 ± 0.5	8.7 ± 1.0	<0.0001

Quantitative data expressed as mean ± SD, except for UAC presented as median (25th, 75th percentiles). Statistics are Chi-2, ANOVA, or Kruskal–Wallis tests. *p* < 0.008 was considered as significant. eGFR: estimated glomerular filtration rate; UAC: urinary albumin concentration; ACE: angiotensin-I-converting enzyme; ARBs: angiotensin receptor blockers.

**Table 2:** Risks of ESKD during follow-up by tertiles of redox genetic risk score.

	ESKD during Follow-Up		Incidence Rate	Adjusted Model 1		Adjusted Model 2	
	No	Yes, *n* (%)	(95% CI)	Hazard Ratio (95% CI)	*p*	Hazard Ratio (95% CI)	*p*
**Tertiles of GRS**							
First tertile	314	23 (6.8)	5.4 (3.6–8.1)	reference		reference	
Second tertile	316	21 (6.2)	4.5 (2.9–6.8)	0.92 (0.50–1.66)	0.78	1.40 (0.73–2.67)	0.31
Third tertile	277	61 (18.1)	13.5 (10.4–17.3)	2.87 (1.77–4.65)	<0.0001	2.60 (1.51–4.48)	0.001

Data expressed as number of participants (and % of incident ESKD). Incidence rates expressed per 1000 person-years. Hazard ratios with 95% confidence intervals (CI) for the second and third tertiles of redox genetic risk score (GRS) versus the first one, computed by Cox regression analysis, adjusted for: Model 1: cohort membership, sex and age; Model 2: model 1 plus duration of diabetes, systolic blood pressure, HbA1c, eGFR, urinary albumin concentration, diabetic retinopathy stages (absent, non-proliferative, proliferative), use of ACE inhibitors or ARBs drugs and tobacco smoking at baseline. *p* < 0.008 was considered as significant.

**Table 3:** Risks of ESKD during follow-up by redox genetic risk score, considered as continuous variable, in each individual cohort.

	ESKD during Follow-Up				
	N (%)	Incidence Rate (95% CI)	Hazard Ratio (95% CI)	*p*	*p* for Interaction *
Pooled cohorts	105 (10.4)	7.8 (6.4–9.4)	1.20 (1.08–1.33)	<0.0001	
Individual cohorts					
SURGENE	17 (5.2)	2.9 (1.8–4.7)	1.49 (1.01–2.21)	0.04	0.49
GENEDIAB	48 (17.6)	15.0 (11.3–20.0)	1.24 (1.04–1.47)	0.02	
GENESIS	40 (9.7)	9.0 (6.6–12.2)	1.25 (1.07–1.46)	0.005	

Data expressed as number of participants (%) and incidence rates (per 1000 persons-years) of ESKD. Hazard ratio with 95% confidence intervals (CI) for each increased risk allele, computed by Cox regression analysis, adjusted for cohort membership (only in the pooled cohorts), sex, age, duration of diabetes, systolic blood pressure, HbA1c, eGFR, urinary albumin concentration, diabetic retinopathy stages (absent, non-proliferative, proliferative), use of ACE inhibitors or ARBs drugs and tobacco smoking at baseline. * Interaction was assessed by including “cohort membership#redox GRS” term in the Cox model. *p* < 0.008 was considered as significant.

## Data Availability

The datasets analyzed during the current study are not publicly available due to consideration of intellectual property, due to many ongoing active collaborations, and due to continuing analyses by the study investigators, but may be available from the first author on reasonable request.

## Supplementary Materials

The following supporting information can be downloaded at: https://www.mdpi.com/article/10.3390/cells11244131/s1, Figure S1. Study flow-chart; Figure S2. Hazard ratios for ESKD by redox genetic risk score; Table S1. Minor allele frequencies of identified polymorphisms included in the GRS in the whole cohorts and in participants included or excluded from the present study; Table S2. Characteristics of participants at baseline according to the incidence of ESKD during follow-up.
