# Supplementary material for: Redox Genetic Risk Score and the Incidence of End-Stage Kidney Disease in People with Type 1 Diabetes
Source: Cells. 2022 Dec 19;11(24):4131. doi: 10.3390/cells11244131 (PMC9777489; doi:10.3390/cells11244131)
Supplement: Supplementary file 1 [file cells-11-04131-s001.zip › cells-1989402-supplementary.pdf]

Redox Genetic Risk Score and the Incidence of End-Stage Kidney Disease in People with Type 1 Diabetes

Kamel Mohammedi, Michel Marre, Samy Hadjadj, Louis Potier, Gilberto Velho

SUPPLEMENTARY MATERIALS

Supplemental Figure S1. Study flow-chart

Supplemental Figure S2. Hazard ratios for ESKD by redox genetic risk score

Supplemental Table S1. Minor allele frequencies of identified polymorphisms included in the GRS in the whole cohorts and in participants included or excluded from the present study

Supplemental Table S2. Characteristics of participants at baseline according to the incidence of ESKD during follow-up

List of Contributors to SURGENE, GENEDIAB, and GENESIS studies

Supplemental Figure S1. Study flow-chart

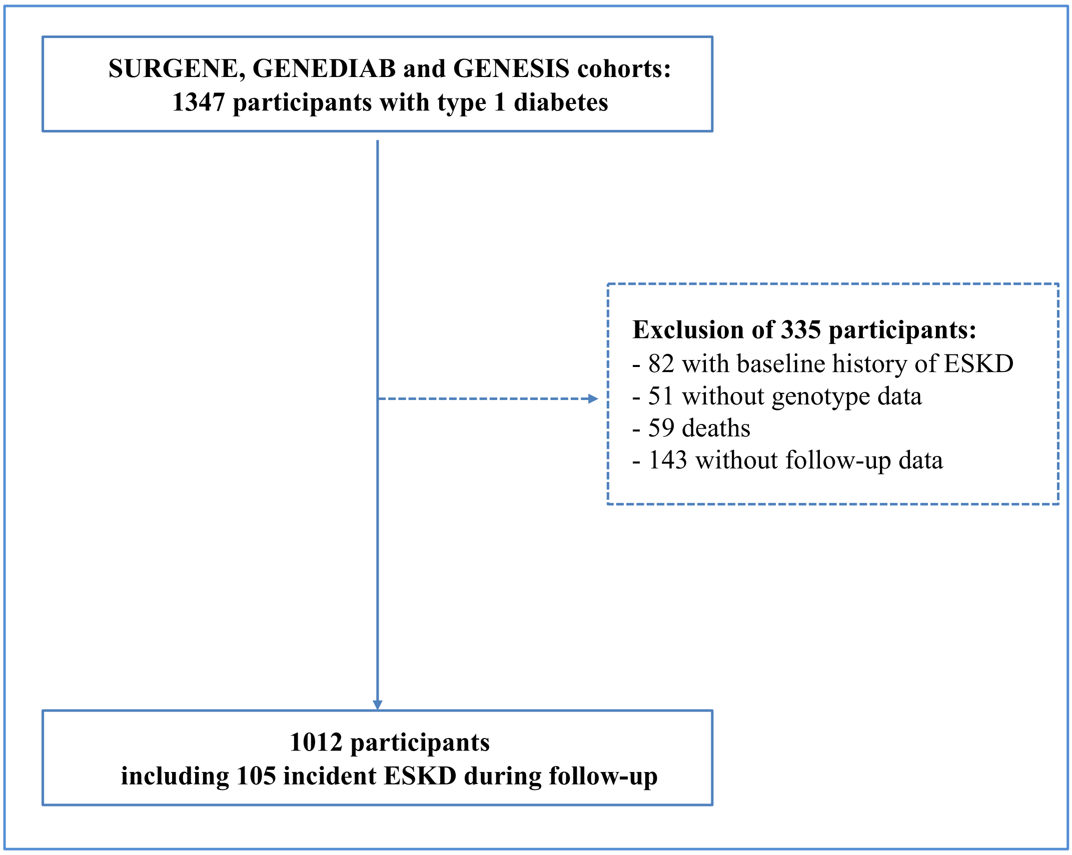

Supplemental Figure S2. Hazard ratios for ESKD by redox genetic risk score

Multi-adjusted hazard ratios (blue solid line) and 95% confidence intervals (red dotted lines) for end-stage kidney disease (ESKD) during follow-up according to redox genetic risk score as a continuous variable with a reference value at 4 (diamond).

Analyses were adjusted for cohort membership, sex, age, duration of diabetes, systolic blood pressure, HbA1c, eGFR, urinary albumin concentration, diabetic retinopathy stages (absent, non-proliferative, proliferative), use of ACE inhibitors or ARBs drugs and tobacco smoking at baseline.

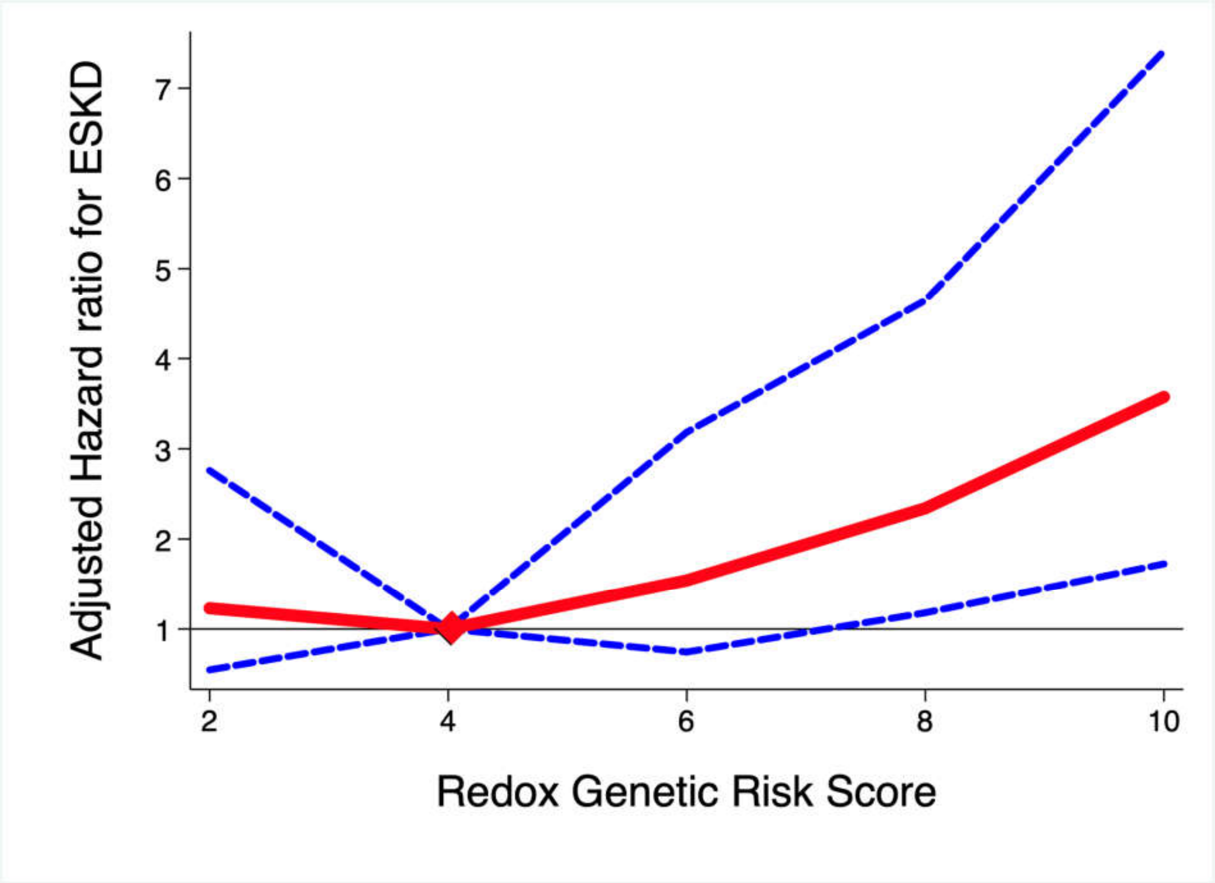

Supplemental Table S1. Minor allele frequencies of identified polymorphisms included in the GRS in the whole cohorts and in participants included or excluded from the present study

		**Minor allele frequencies**		
		**All** (N=1347)	**Excluded participants** (N=335)	**Included participants** (N=1012)
**Genes**	**SNPs**			
CAT	rs2420388	0.240	0.260	0.240
GPX1	rs9818758	0.180	0.180	0.180
GPX4	rs713041	0.470	0.490	0.460
SOD2	rs4880	0.470	0.470	0.470
SOD3	rs2270224	0.500	0.500	0.500
CYBA	rs11076692	0.510	0.500	0.510

Supplemental Table S2. Characteristics of participants at baseline according to the incidence of ESKD during follow-up

	ESKD during follow-up		p
	No, n (%)	Yes, n (%)	
N (%)	907 (89.6)	105 (10.4)	
Cohort membership, n (%)			<0.0001
SURGENE	310 (34.2)	17 (16.2)	
GENEDIAB	225 (24.8)	48 (45.7)	
GENESIS	372 (41.0)	40 (38.1)	
Sex (women), n (%)	402 (44.3)	51 (48.6)	0.69
Age, years	39.8 ± 13.0	40.0 ± 11.1	0.89
Duration of diabetes, years	22.7 ± 11.6	24.9 ± 9.4	0.06
Body mass index, kg/m^2	24 ± 3	24 ± 4	0.53
Systolic blood pressure, mmHg	129 ± 17	148 ± 20	<0.0001
Diastolic blood pressure, mmHg	75 ± 10	85 ± 12	<0.0001
HbA1c, %	8.8 ± 1.8	9.3 ± 2.1	0.007
HbA1c, mmol/mol	72 ± 20	78 ± 23	
eGFR, ml/min/1.73m^2	94 ± 24	54 ± 32	<0.0001
Categories of eGFR, n (%)			<0.0001
≥ 60 ml/min/1.73 m^2	834 (91.9)	37 (35.2)	
< 60 ml/min/1.73 m^2	73 (8.1)	68 (64.8)	
UAC, mg/l	10 (5, 38)	1033 (281, 2090)	<0.0001
Categories of UAC, n (%)			<0.0001
Normoalbuminuria (<30 mg/l)	642 (70.8)	9 (8.6)	
Microalbuminuria (30 – <300 mg/l)	179 (19.7)	18 (17.1)	
Macroalbuminuria (≥300 mg/l)	86 (9.5)	78 (74.3)	
Use of ACE inhibitors or ARBs, n (%)	252 (27.9)	70 (66.7)	<0.0001
Use of any antihypertensive drug, n (%)	311 (34.4)	88 (83.8)	<0.0001

Use of lipid-lowering drugs, n (%)	37 (3.9)	1 (0.9)	0.11
Tobacco smoking, n (%)			0.002
Former smokers	88 (9.7)	13 (12.4)	
Current smokers	173 (19.1)	26 (24.8)	
History of diabetic retinopathy			<0.0001
Absent	201 (22.1)	1 (1)	
Non-proliferative	387 (42.7)	28 (26.6)	
Proliferative	319 (35.2)	76 (72.4)	
History of myocardial infarction, n (%)	31 (3.4)	5 (4.8)	0.50
History of stroke, n (%)	13 (1.4)	5 (4.8)	0.04
Redox genetic risk score	6.3 ± 2.1	7.2 ± 2.2	0.0001

Quantitative data expressed as mean±SD, except for UAC presented as median (25th, 75th percentiles). Statistics are Chi-2, ANOVA, or Wilcoxon tests. P<0.008 was significant.

eGFR: estimated glomerular filtration rate; UAC: urinary albumin concentration; ACE: angiotensin-I-converting enzyme; ARBs: angiotensin receptor blockers.

List of Contributors to SURGENE, GENEDIAB, and GENESIS studies

Clinical investigators at baseline by alphabetical order of enrollment centers

All cities are in France, except for Liège, in Belgium:

Albert Fournier, Jean-Daniel Lalau (*Centre Hospitalier Universitaire d'Amiens*); Béatrice Bouhanick, Line Godiveau, Michel Marre, Vincent Rohmer (*Centre Hospitalier Universitaire d'Angers*); Jean-Raymond Attali, Patrick Miossec (*Assistance Publique des Hôpitaux de Paris, Hôpital de Bondy*); Henri Gin, Vincent Rigalleau (*Centre Hospitalier Universitaire de Bordeaux*); Isabelle Cerf, Guillaume Charpentier, Isabelle Petit, Jean-Pierre Riveline (*Centre Hospitalier de Corbeil-Essonne*); Bertrand Godeau, Zoubida Kahal, Dominique Simon (*Assistance Publique des Hôpitaux de Paris, Hôpital de Créteil*); Daniel Cordonnier, Serge Halimi (*Centre Hospitalier Universitaire de Grenoble*); Pierre-Jean Lefebvre, Nicolas Paquot, André Scheen, Laurent Weekers (*Centre Hospitalier Universitaire de Liège*); Pierre Fontaine, Gaëtan Prevot (*Centre Hospitalier Régional Universitaire de Lille*); François Berthezene, Fabrice Bonnet, Maurice Laville, Jean-Pierre Fauvel, Charles Thivolet (*Assistance Publique des Hôpitaux de Lyon*); Bertrand Dussolle, Philippe Vague (*Assistance Publique des Hôpitaux de Marseille*); Patrick Giraud (*Clinique Pont de Chaume, Montauban*); Jacques Bringer, Florence Galtier, Michel Rodier (*Centres Hospitaliers Universitaires de Montpellier et Nîmes*); Pierre Drouin, Laurent Dusselier, Thérèse Crea, Bruno Guerci, Michèle Kessler (*Centre Hospitalier Universitaire de Nancy*); Lucy Chaillous, Bernard Charbonnel (*Centre Hospitalier Universitaire de Nantes*); Hamid Boukersi, Françoise Defrance, Etienne Larger, Michel Marre, Ronan Roussel (*Assistance Publique des Hôpitaux de Paris, Hôpital Bichat-Claude Bernard*); Gérard Slama, Agnès Sola (*Assistance Publique des Hôpitaux de Paris, Hôpital de l'Hôtel Dieu*); André Grimaldi, Agnès Heurtier, Caroline Sert (*Assistance Publique des Hôpitaux de Paris, Hôpital de La Pitié Salpétrière*); Jean-Pierre Grunfeld (*Assistance Publique des Hôpitaux de Paris, Hôpital Necker*); Ahmed Bouallouche, Jean-François Gautier, Pierre-Jean Guillausseau, Hervé Leblanc, Philippe Passa (*Assistance Publique des Hôpitaux de Paris, Hôpitaux Saint-Louis et Lariboisière*); Samy Hadjadj, Richard Maréchaud, Anne Muller, Florence Torremocha (*Centre Hospitalier Universitaire de Poitiers*); Jacques Chanard, Jean-Paul Melin (*Centre Hospitalier Universitaire de Reims*); Hubert Allannic, Jean-Yves Poirier (*Centre Hospitalier Universitaire de Rennes*); Christian Charasse (*Centre Hospitalier de Saint-Brieuc*); Bernard Bauduceau, Lyse Bordier, Hervé Mayaudon (*Hôpital d'Instruction des Armées Bégin, Saint-Mandé*); Marie-Pierre Arpin-Bott (*Centre*

Hospitalier Universitaire de Strasbourg); Hélène Hanaire, Pierre Gourdy, Henri Sackmann, Jean-Pierre Tauber (*Centre Hospitalier Universitaire de Toulouse*); Odile Verier-Mine (*Centre Hospitalier de Valenciennes*).
